# Tunable characteristics of low-frequency bandgaps in two-dimensional multivibrator phononic crystal plates under prestrain

**DOI:** 10.1038/s41598-021-87904-6

**Published:** 2021-04-16

**Authors:** Hai-Fei Zhu, Xiao-Wei Sun, Ting Song, Xiao-Dong Wen, Xi-Xuan Liu, Jin-Shan Feng, Zi-Jiang Liu

**Affiliations:** 1 School of Mathematics and Physics, Lanzhou Jiaotong University, Lanzhou, 730070 China; 2 Department of Physics, Lanzhou City University, Lanzhou, 730070 China

**Keywords:** Structure of solids and liquids, Composites, Acoustics

## Abstract

In view of the influence of variability of low-frequency noise frequency on noise prevention in real life, we present a novel two-dimensional tunable phononic crystal plate which is consisted of lead columns deposited in a silicone rubber plate with periodic holes and calculate its bandgap characteristics by finite element method. The low-frequency bandgap mechanism of the designed model is discussed simultaneously. Accordingly, the influence of geometric parameters of the phononic crystal plate on the bandgap characteristics is analyzed and the bandgap adjustability under prestretch strain is further studied. Results show that the new designed phononic crystal plate has lower bandgap starting frequency and wider bandwidth than the traditional single-sided structure, which is due to the coupling between the resonance mode of the scatterer and the long traveling wave in the matrix with the introduction of periodic holes. Applying prestretch strain to the matrix can realize active realtime control of low-frequency bandgap under slight deformation and broaden the low-frequency bandgap, which can be explained as the multiple bands tend to be flattened due to the localization degree of unit cell vibration increases with the rise of prestrain. The presented structure improves the realtime adjustability of sound isolation and vibration reduction frequency for phononic crystal in complex acoustic vibration environments.

## Introduction

Most of the noise in our life is distributed in the low-frequency range of 20–200 Hz. As low-frequency noise brings great harm to human health, industrial production, bridge construction, etc., the design of structures to control low-frequency noise and vibration from the propagation path through structural design and optimization has become a research hotspot^1–4^. Over the past three decades, phononic crystal (PnC) has attracted widespread attention for their excellent ability to control the propagation of elastic waves and its waveguide characteristics^5–8^. PnC is an artificial composite material with elastic constant and density periodic distribution^9,10^. The concept of PnC was first proposed by Kushwaha et al*.*^11^ in 1993. Due to the periodicity of the structure, the elastic wave in a specific frequency range cannot propagate in it, that is, there is an elastic wave bandgap (BG). Up to now, there exist two mature mechanisms for the BG formation of PnC, Bragg scattering mechanism and local resonance mechanism^12,13^. The former emphasizes the size effect of periodic structure on waves, viz., the destructive interference occurs on rigid boundaries. Nevertheless, the resulting elastic wave BG is usually limited to the high frequency range. The latter is known as acoustic metamaterial, which mainly emphasizes the motion mode of the single primitive cell, that is, the resonance characteristic of scatterers play a dominant role. The acoustic energy is localized in the unit cell due to the reverse vibration of the matrix as well as the local resonator and finally the BG is opened in the low frequency range. By combining the BG characteristics and defect state characteristics of PnCs or introducing functional materials and designing special structures, etc., PnCs can have a wider application prospect in acoustic device design and acoustic signal processing, such as the optimization of acoustic device design including sound insulation and vibration reduction structure^14^, acoustic waveguide^15^ as well as acoustic filter^16^ and improvement of acoustic collimation^17^, acoustic focusing^18,19^, acoustic energy recovery^20,21^ as well as other technologies.

For the prevention and control of low-frequency noise and vibration, the existing researches in PnC field mainly focus on passive optimization and active control. The former optimizes the model structure mainly by considering the symmetry of the structure, the selection of materials and the arrangement of structural components, while the latter satisfies the requirements of sound isolation and vibration reduction under complex acoustic vibration environment by active realtime control of BG. In recent years, some works have obtained a wide BG in the low-frequency range by combining the two BG formation mechanisms and optimized the BG by changing geometric parameters^22–24^. Coffy et al*.*^25^ designed the strip PnC by combining the two mechanisms and optimized the geometric parameters of the model. Finally, the relative bandwidth of the band structure of the model, namely, the ratio of the BG width to the BG center frequency, reached 138%. But the BGs did not generate in the low-frequency range of 20–200 Hz. A new type of local resonance composite-type PnC structure was presented by Zhang and Wu^26^. By changing the structural parameters, a resonant BG with a bandwidth of more than 60% was opened in the frequency range of 0–200 Hz. Especially, the BG starting frequency is as low as 18 Hz, but the low-frequency BG still needs to be widened. Zhang and Han^27^ further designed a PnC plate with periodic holes and widened the BG by changing the geometric parameters of the model. Finally, the structure generated a BG with a width of 53.2% in the frequency range of 0–500 Hz.

The acoustic properties of PnCs are almost unchanged after processing and molding, although the low-frequency BG can be obtained by optimizing the geometric parameters of PnCs. Nevertheless, the noise frequency is variable in the scenes along rail transit and highways, etc. Consequently, it is necessary to design a PnC possessing the flexible realtime adjustability and the favorable low-frequency noise attenuation characteristics simultaneously. Research shows that external excitation including mechanical load^28,29^, electric field^30,31^, magnetic field^32^, temperature^33^ are able to change the elasticity and other properties of materials to realize the realtime reversible control of BG. Among the relevant mechanisms of external excitation to adjust the BG of PnCs, the pressure field regulation mechanism is an ideal application method due to the high availability of materials and the easy realization of regulation methods. Bertoldi and Boyce^34^ investigated the influence of finite deformation on elastic wave propagation in periodic hyperelastic materials by finite element method (FEM). The results show that structure instabilities can be introduced into this material by external mechanical load. At this point, the equilibrium configuration of the structure will change greatly by adding a small increment and the BG can be adjusted by controlling the degree of finite deformation. Shan et al*.*^35^ further introduced periodic holes into PnCs composed of hyperelastic materials. Three different buckling modes namely vertical deformation, horizontal deformation and equi-biaxial deformation were triggered by changing the loading direction during the progress of compression loading, thus changing the deformation mode as well as degree of the model and finally achieve the effect of adjusting the BG. In the above experiments, BG control is realized by applying large deformation to the model to change the material characteristics, which limits the practical application of this type of PnCs. Huang et al*.*^36,37^ studied the effect of large deformation and prestretch on the BG of PnC made of hyperelastic material by FEM, respectively. Among them, the prestretch strain can tune the frequency range of BG almost without changing the geometric size of the model, which broadens the application range of this type of PnCs.

Applying mechanical load to control the BG of PnC composed of soft material provides a new idea and method for realtime regulation of BG. Nevertheless, the adjustable BG frequency range of the proposed model cannot better meet the actual requirements of vibration and noise reduction, especially the low-frequency noise between 20 and 200 Hz. As such, an adjustable two-dimensional novel PnC plate with single-sided columns and periodic holes is proposed. The BG characteristic of the PnC plate are calculated by FEM. Based on the local resonance mechanism, the generation mechanism of the low-frequency BG of the structure is analyzed. Considering the limitations of lattice constant *a* in practical applications, the low-frequency BGs are opened by selecting excellent key parameters. On this basis, equal biaxial prestretch strain is further introduced to investigate the realtime tunability of low-frequency BG. The reason for generating multiple local resonance BGs in the low-frequency range is explained by analyzing the eigenmodes. It is found that increasing the prestretch strain can improve the localization degree of unit cell vibration in a certain frequency range, thus reducing the speed of sound energy transfer as well as making the band curve tend to be flat and finally open a plurality of local resonance BGs in the low-frequency range.

## Models design and calculation method

**Figure 1:**
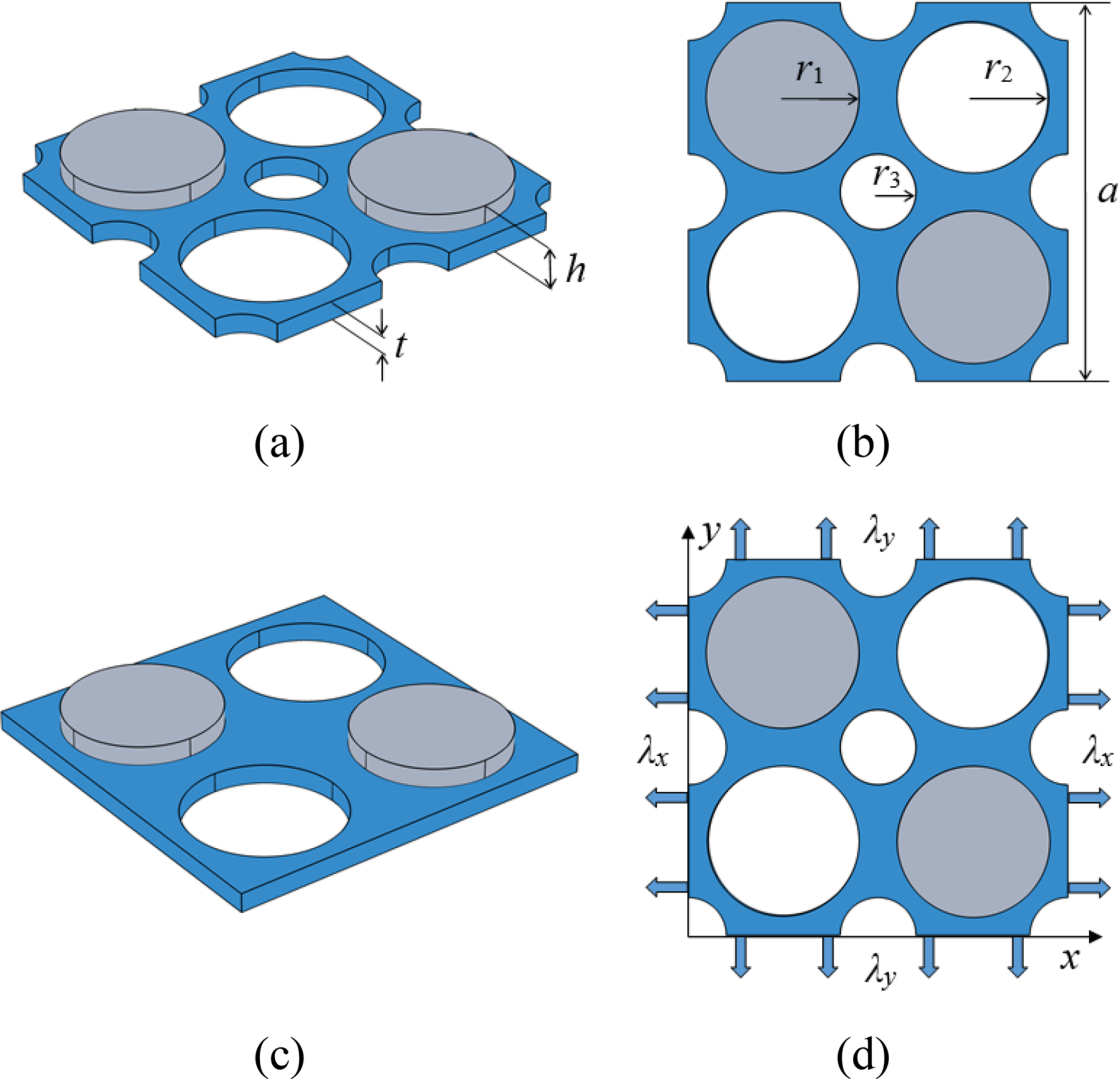
Unit cell of the PnCs. The schematic and the top view of new designed PnC plate are shown in (**a**) and (**b**), respectively. (**c**) corresponds to the single-sided pillar-type PnC plate with holes. The boundary conditions imposed on the unit cell are shown in (**d**).

In this work, a new designed two-dimensional multivibrator PnC plate is proposed. The introduction of periodic holes leads to the existence of multiple vibrators in the matrix plate and the coupling of vibration modes among the multiple vibrators is conducive to widen the BG. Diagonal holes and single-sided diagonal columns are introduced into the porous matrix plate. By adjusting the in-plane and out-of-plane symmetry of the structure, new local resonance BGs are opened in the low-frequency range, thereby breaking through the low-frequency limit. The matrix plate is made of pressure sensitive soft silicone rubber, which facilitates the modulation of pressure field. In parallel, considering the cost of materials and the impedance effect caused by the high impedance difference of the material, lead is used as the scatterer material, which is easy to open the wide BG in the low-frequency range. Figure 1a,b are new designed porous single-sided column PnC plates with lattice constant *a*, plate thickness *t*, column height *h*, column radius *r*_1_, diagonal hole radius *r*_2_ and periodic hole radius *r*_3_. The BG of a traditional single-sided cylindrical PnC plate was calculated as a comparison and its unit cell diagram is shown in Fig. 1c^27^. When external loads are applied to the PnC structure, the boundary conditions need to be applied to the matrix plate of unit cell along the *x* and *y* directions, as shown in Fig. 1d.

**Figure 2:**
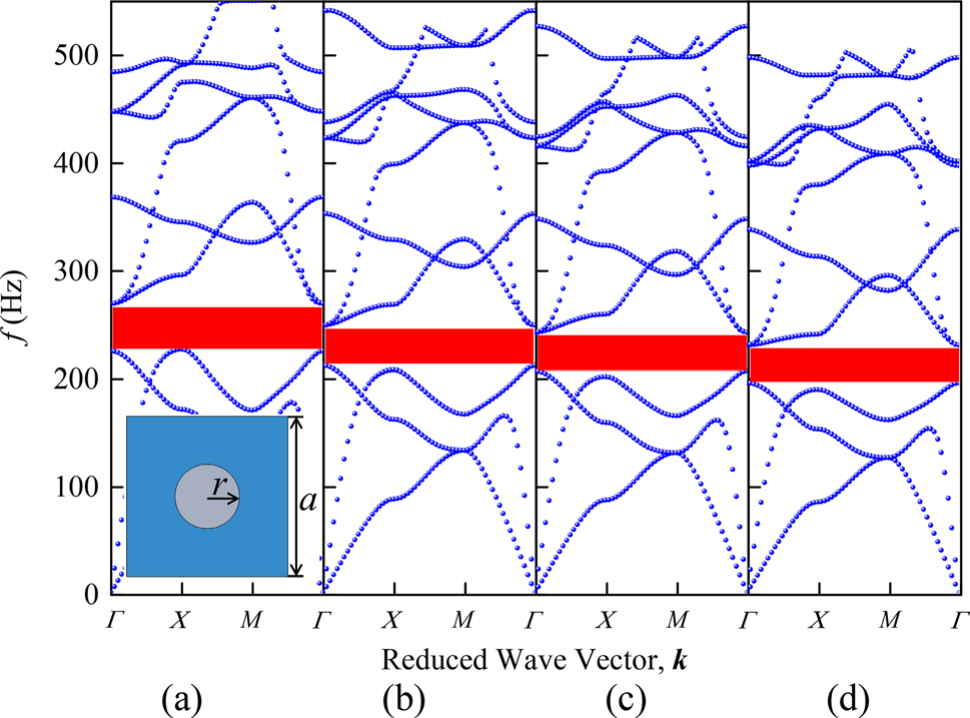
The band structure calculated by PWEM are shown in (**a**–**c**) when the number of plane wave is taken as 441, 1681 and 3721, respectively. The calculation results of FEM are given in (**d**). The schematic of the unit cell of the PnC used in the comparison calculation is shown in the inset.

The inset in Fig. 2 is a two-dimensional PnC composed of cylindrical scatterers embedded in square matrix plate, where gray parts represent lead columns and blue parts represent silicone rubber matrix plates. The PnC has infinite size in *z* direction. The lattice constant *a* = 20 mm and cylinder radius *r* = 4 mm. For the same calculation model, the choice of calculation method directly affects the calculation efficiency and the accuracy of the results. So we calculated the band structure of the model shown in Fig. 2 by using the plane wave expansion method (PWEM) and FEM, respectively. It can be seen from Fig. 2a–c that the BG frequency tends to low-frequency with increasing plane wave number, the BG convergence property increases accordingly and gradually approaches the calculation result of the FEM. But its calculation time also increases greatly. When the plane wave number is taken as 3721, the calculation time of PWEM is about 1 h and 20 min, which is longer than the time of 1 min and 39 s of the FEM under the refined calculation mesh in Fig. 2d.

In view of the complexity of the model designed in this work and considering the problems of convergence property and calculation efficiency, the FEM is adopted to calculate the BG characteristic of the model. The study of finite deformation in prestrain part is on the basis of the general nonlinear elasticity theory in finite deformed structure^37–39^. Huang et al*.*^37^ also calculated the BG characteristics of two-dimensional double-sided cylindrical PnC plates under different prestrain based on the general nonlinear elasticity theory by using the FEM. They found that the frequency calculated by the finite element software COMSOL Multiphysics is in good agreement with the calculated value of ABAQUS, which verified the correctness and accuracy of FEM simulation.

The FEM provides great freedom in the choice of dispersion degree. In the process of solving, the mesh should be divided appropriately according to the complexity of the object to be solved, so as to ensure the accuracy of the calculation results and improve the efficiency of the solution. The accuracy of our results is guaranteed by mesh refinement. The difference between the FEM used to solve the BG characteristics of PnCs and the traditional engineering problem is that the former introduces Bloch displacement waves and periodic boundary conditions during the solution process. Then the displacement boundary condition can be obtained:

1 $$ {\varvec{u}}\left( {{\varvec{r}} + {\varvec{R}}_{n} } \right) = {\varvec{u}}\left( {\varvec{r}} \right)e^{{i\left( {{\varvec{k}} \cdot {\varvec{R}}_{n} } \right)}} , $$

where ***r*** is the position vector of the boundary node, ***k*** represents the wave vector, ***R***_*n*_ is the lattice vector for the PnC and ***u***(***r***) is used to represent a periodic vector function.

We can solve the eigenvalue Eq. (2) by using COMSOL Multiphysics 5.3^40^,

2 $$ \left( {{\mathbf{K}} - \omega^{2} {\mathbf{M}}} \right){\mathbf{U}} = 0, $$

where **U** is the node displacement, **K** and **M** represent the stiffness matrix and mass matrix of the unit cell, respectively. The dispersion relation and eigenmodes can be obtained by using the eigenvalue from output.

**Figure 3:**
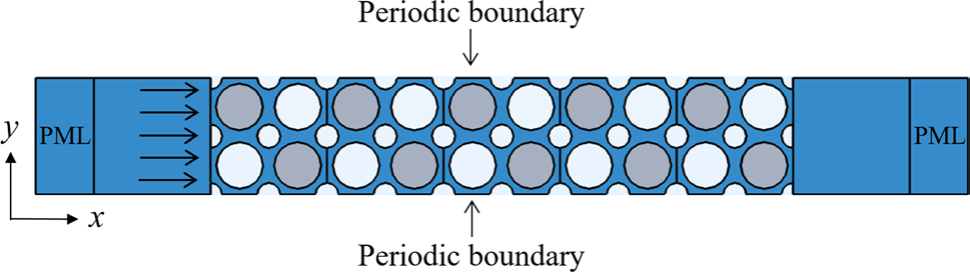
Finite structure for the calculation of transmission loss.

Figure 3 shows a finite 1 × 5 period structure used in calculating transmission loss. The average displacement excitation *d*_in_ and response *d*_out_ are applied to the left and right end of the periodic plate in the *x* direction, respectively. To avoid the energy reflection of the elastic wave on the boundary from affecting the calculation results, two perfectly matched layers (PMLs) with a length of 0.01 m are used in the *x* direction. The periodic boundary is used in the *y* direction. Transmission loss *T* is defined as:

3 $$ T = 20\log \frac{{d_{{{\rm out}}} }}{{d_{{{\rm in}}} }}. $$

In this work, the displacement boundary condition is applied to the matrix plate of unit cell along the *x* and *y* directions in the prestrain research process, respectively. The periodic displacement boundary condition can be described by Eq. (4) ^37^,

4 $$ \left. {\begin{array}{*{20}l} {\frac{U(a,y,z) - U(0,y,z)}{a} = 1 + \lambda_{x} ,} \hfill \\ {V(a,y,z) = V(0,y,z),} \hfill \\ {W(a,y,z) = W(0,y,z),} \hfill \\ \end{array} } \right\}\;P_{x} \left. {\begin{array}{*{20}l} {\frac{V(a,y,z) - V(0,y,z)}{a} = 1 + \lambda_{y} ,} \hfill \\ {U(a,y,z) = U(0,y,z),} \hfill \\ {W(a,y,z) = W(0,y,z),} \hfill \\ \end{array} } \right\}\;P_{y} $$

where *U*, *V* and *W* are the position vector components of the points at the unit cell boundary in the *x*, *y* and *z* directions, respectively, *P*_*x*_ and *P*_*y*_ represent the two boundaries of the unit cell in the *x* and *y* direction, while *λ*_*x*_ and *λ*_*y*_ are nominal strains along the *x* and *y* directions, respectively. *λ*_*x*_ and *λ*_*y*_ represent normalized dimensionless parameter.

## Results and discussion

### Analysis of bandgap mechanism

The novel PnC model proposed in this work selects silicone rubber as the matrix material and lead as the columnar scatterer material. The corresponding material parameters of silicone rubber are taken as density *ρ* = 1300 kg m^−3^, Young modulus *E* = 1.175 × 10^5^ Pa and Poisson’s ratio *υ* = 0.46875, respectively. For lead, *ρ* = 11,600 kg m^−3^, *E* = 4.08 × 10^10^ Pa and *υ* = 0.3691. The geometrical parameters are assumed as follows. The lattice constant *a* = 20 mm, plate thickness *t* = 1 mm, column radius *r*_1_ = 4 mm, diagonal hole radius *r*_2_ = 4 mm, periodic hole radius *r*_3_ = 2 mm and column height *h* = 2 mm.

**Figure 4:**
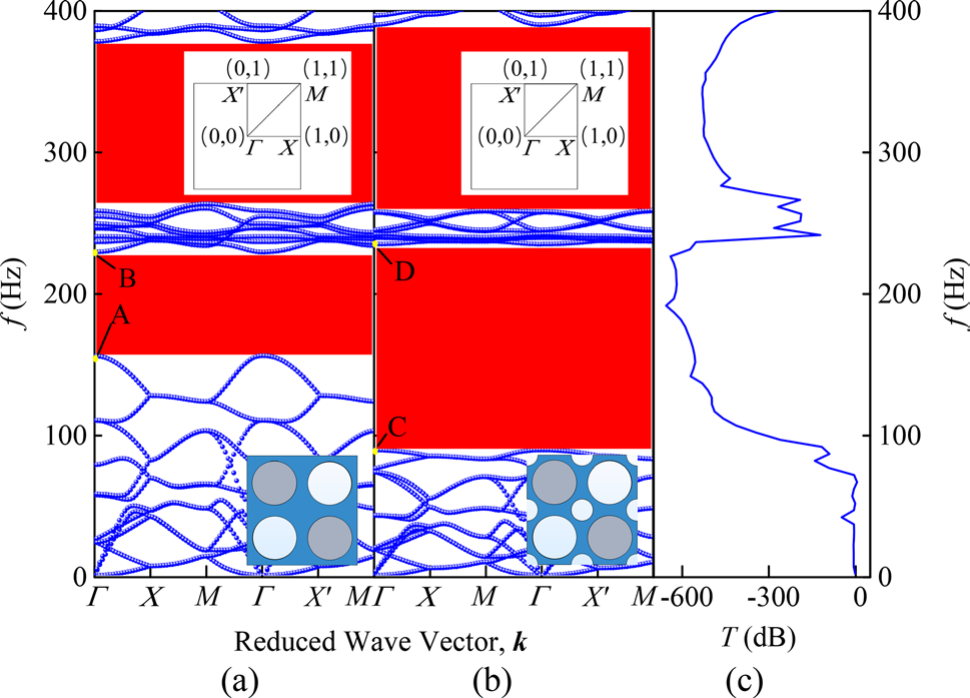
Panels (**a**) and (**b**) correspond to schematic diagrams of band structure of single-sided pillar-type PnC plate with holes and novel PnC plate. The inset shows the top view of the two models and the irreducible Brillouin zone of the corresponding unit cell, respectively. The transmission characteristic of novel PnC plate is shown in (**c**).

Comparing the band structure diagrams in Fig. 4, the novel PnC plate has a lower starting frequency of the first BG than the traditional PnC plate with the same parameters and the BG in the frequency range of 0–400 Hz accounts for 71%, which is better than 47% of the traditional structures. The transmission characteristics in Fig. 4c verifies the accuracy of the band structure.

**Figure 5:**
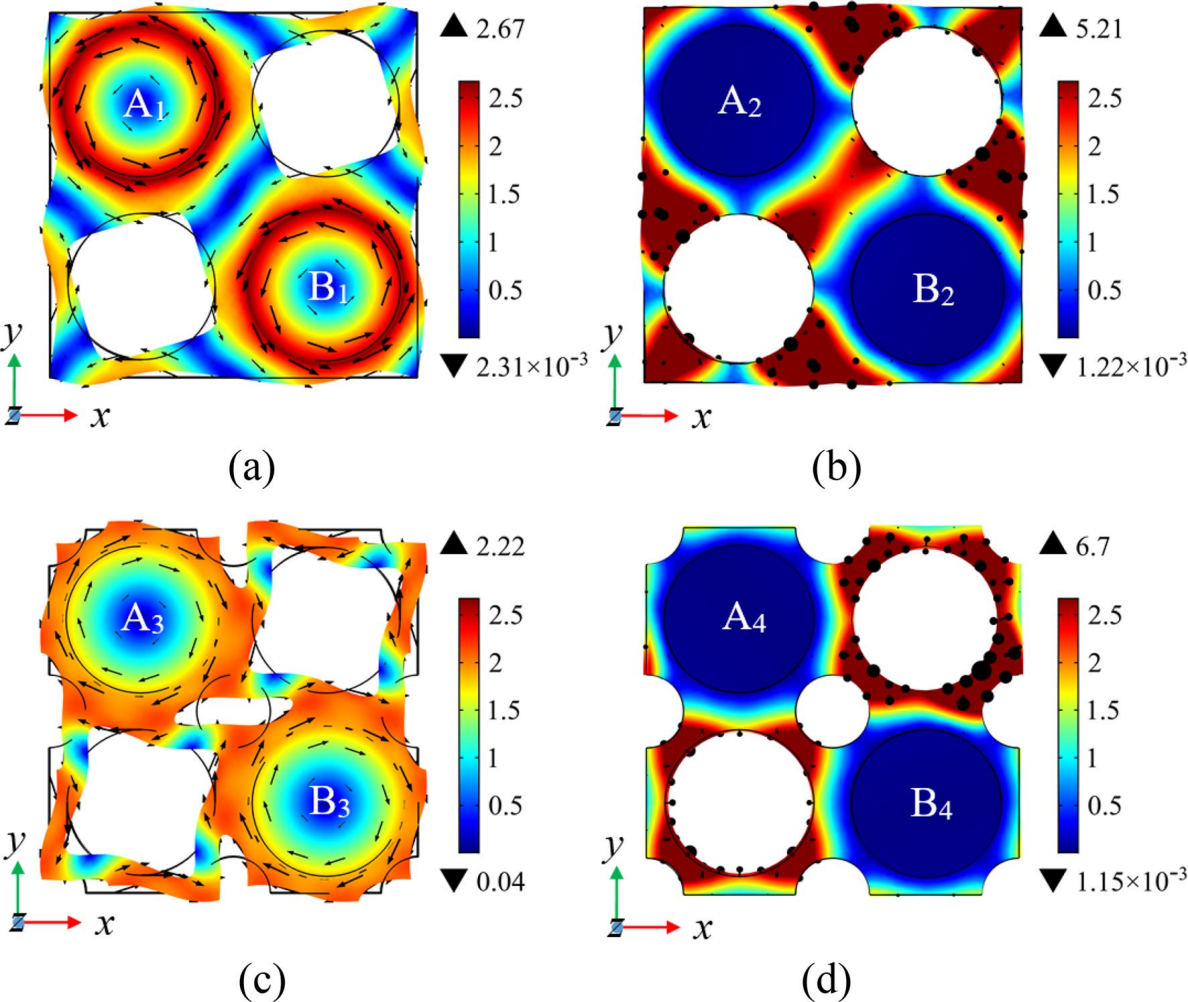
The eigenmodes shapes in the low frequency range. The eigenmodes shapes at A, B, C and D in the band structure of Fig. 4 are shown in (**a**–**d**), respectively.

To analyze the generation mechanism of the low-frequency BG in the proposed structure, Fig. 5a–d show the modes corresponding to the four points A, B, C and D in Fig. 4, respectively. As shown in Fig. 5a,c, the eigenmodes shapes corresponding to the 12th band of the comparison model and the novel model are mainly composed of in-plane torsional vibrations of cylinders and the reverse torsional vibration of the matrix. Nevertheless, due to the small amplitude of the matrix of comparison model, it implies difficulties to resonate with the cylinder. When periodic holes are introduced into the matrix, the matrix and the cylinder form anti-resonance. At this time, the resonance mode of the scatterer forms a resultant force in the *x* and *y* directions and is coupled with the long traveling wave in XY mode in the matrix. The energy carried by the traveling wave is continuously consumed and gradually decreases in the coupling process, which causes the slowdown of transfer speed of elastic wave energy at corresponding frequency. Due to the energy consumption of traveling waves, the band curve tends to be flattened, further expanding the local resonance BG.

In the meantime, on account of the introduction of periodic holes, a plurality of vibrators appears in the structure, thus the expansion of the low-frequency BG can also be regarded as the coupling of local resonance modes among multiple vibrators. The part with larger amplitude on the plate is equivalent to a mass block, whose mass is equivalent mass. The part with smaller amplitude is equivalent to a spring, whose stiffness is equivalent stiffness. The eigenfrequency of the system can be calculated by the following formula.

5 $$f = \frac{1}{{2\uppi }}\sqrt {\frac{k}{m}} ,$$

where *m* is the equivalent mass and *k* represents the equivalent stiffness. By observing Fig. 5a,c, the equivalent mass of the vibrating portion increases and the stiffness of the equivalent spring decreases when we introduce the periodic holes, resulting in a significant decrease in the corresponding frequency of the 12th band.

The eigenmodes in Fig. 5b,d are mainly composed of out-of-plane translational vibration of the matrix plate along the* z* direction. The plate not connected to the column, i.e., the plates in red around the diagonal holes in Fig. 5b,d, can be regarded as a mass block. The vibrating part of the plate connected to the column, that is, the plate in blue around the column in Fig. 5b,d, can be regarded as a spring. As can be seen from Fig. 5b,d that the equivalent mass of the mass block decreases after the periodic holes are introduced, give rise to an increase in the corresponding frequency of the 13th band. Nevertheless, the equivalent spring stiffness decreases because of the connection between the plate regarded as a spring and the adjacent plate becomes narrower, so the increase in amplitude is limited.

What we can learn from the above analysis is that the introduction of periodic holes causes a certain degree of coupling between the resonance mode of the scatterer and the long traveling wave in XY mode in the matrix, resulting in the generation of BGs. For the BG produced by local resonance, the BG width is mainly determined by the degree of localization in the local resonance mode and its coupling effect with the traveling wave in the matrix. Within a certain range, the higher the localization degree of the local resonance mode or the stronger its coupling effect with traveling waves, the larger the bandwidth.

### Optimization of geometric parameters

On the basis of considering the engineering requirements of good stability, high bearing capacity and easy machining, the parameters such as column height *h*, periodic hole radius *r*_3_, column radius *r*_1_ and diagonal hole radius *r*_2_ are optimized, so as to obtain better low-frequency BG characteristics by selecting excellent key parameters.

**Figure 6:**
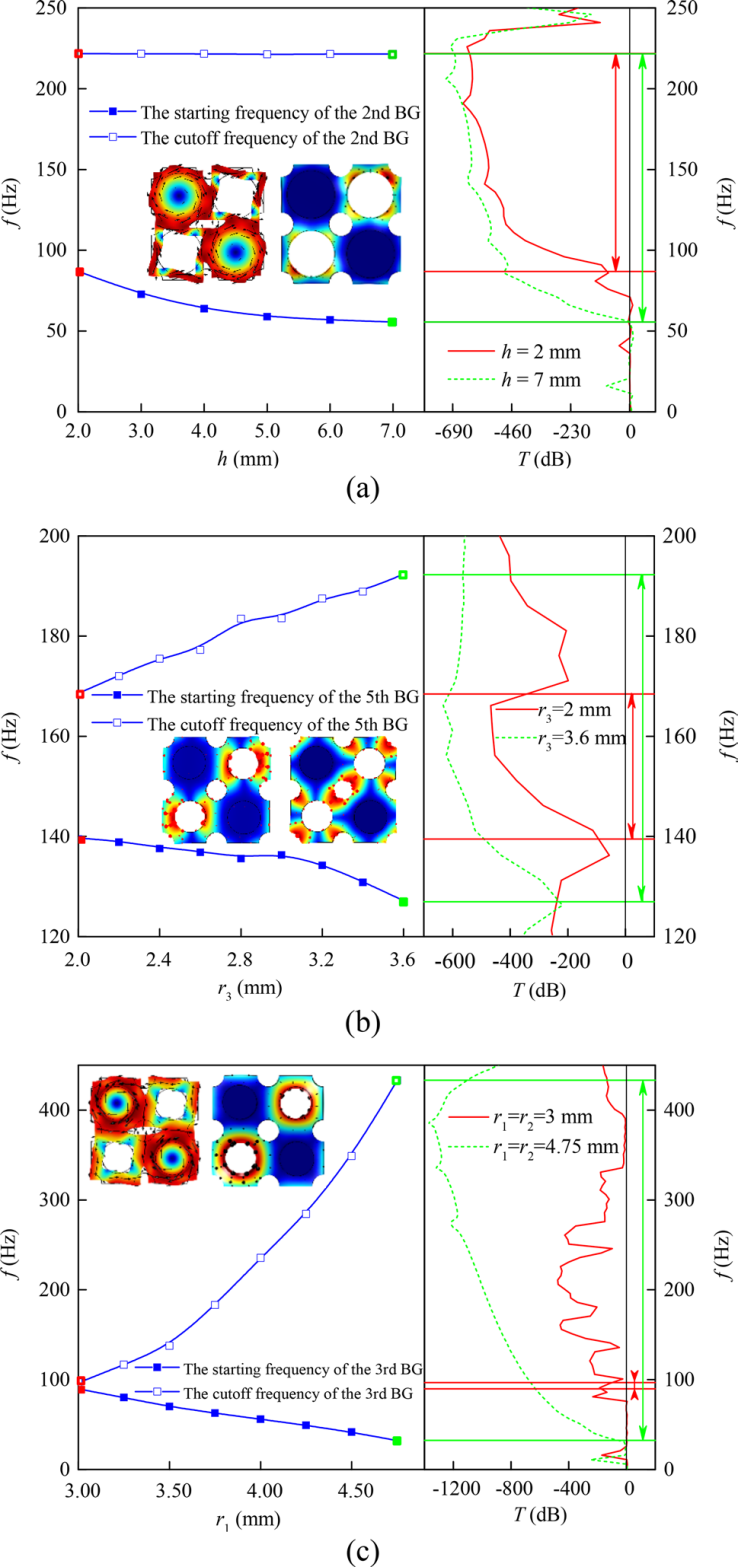
BG characteristics of PnC plate with (**a**) different *h*, (**b**) different *r*_3_ and (**c**) different *r*_1_ and *r*_2_.

Firstly, we analyzed the influence of column height *h* on the structural BG. In the calculation, the geometric parameters are taken as *r*_1_ = 4 mm, *r*_2_ = 4 mm and *r*_3_ = 2 mm. The insets in Fig. 6a show the eigenmodes shapes corresponding to the starting and cutoff frequency of the 2nd BG when *h* is taken as 2 mm, respectively. The former is the in-plane torsion mode, while the latter is mainly the out-plane translational vibration of the matrix plate. As shown in Fig. 6a, the influence of the column height *h* on the starting frequency is relatively obvious, while has little effect on the cutoff frequency. From Eq. (5), the equivalent mass of the scatterer increases and the starting frequency decreases with the increase of column height *h*. The cutoff frequency is determined by the vibration of the connecting plate, so the column height has little influence on the BG cutoff frequency.

BG characteristics of PnC plate with different period hole radii are given in Fig. 6b. In the calculation, *r*_1_ = *r*_2_ = 3 mm and *h* = 7 mm. The change of the starting frequency and the cutoff frequency is shown in Fig. 6b. The insets show the eigenmodes shapes corresponding to the starting and cutoff frequency of the 5th BG when *r*_3_ is taken as 2 mm, respectively. When the radius of the periodic hole increases, the equivalent stiffness of the matrix plate which is regarded as a spring decreases, so does the starting frequency. In parallel, the equivalent mass of the matrix, which is regarded as the mass block, decreases and then the cutoff frequency rises. The attenuation amplitude of transmission characteristics of PnCs also increases.

Finally, the influence of the column radius *r*_1_ and the diagonal hole radius *r*_2_ on the structural BG is analyzed. In the calculation, *r*_1_ and *r*_2_ are taken the same value and *r*_3_ = 2 mm, *h* = 7 mm. The calculation results are shown in Fig. 6c. The insets show the eigenmodes shapes corresponding to the starting and cutoff frequency of the 3rd BG when *r*_1_ and *r*_2_ are taken as 3 mm, respectively. The former is the in-plane torsion mode and the latter is the out-plane translational vibration of the matrix plate. With the increase of *r*_1_ and *r*_2_, the cutoff frequency of BG increases from 96.7 Hz to 433.2 Hz, the starting frequency decreases from 89.7 Hz to 32.3 Hz, the BG widens greatly and the width increases by nearly 60 times. The attenuation amplitude of transmission characteristics of PnCs also increases. According to Eq. (5), when the radius of the column increases, the equivalent mass of the scatterer increases and the starting frequency decreases. The equivalent mass of the plate around the diagonal hole decreases with the increase of the diagonal hole radius and the cutoff frequency increases accordingly.

In summary, the optimization of the low-frequency BG can be realized by increasing the column height to change the out-of-plane asymmetry of the PnC structure and by increasing the radii of diagonal holes and periodic holes to reduce the stiffness of the matrix plate. The above geometric parameters have different effects on the BG width.

### Prestrain study

**Figure 7:**
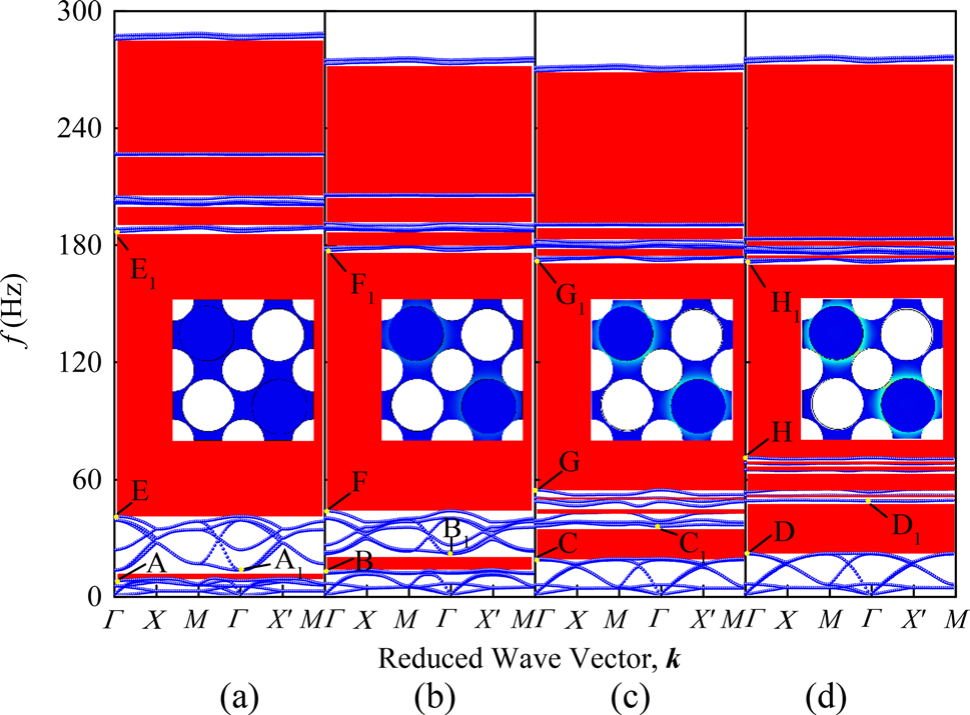
Band structure of PnC plate under different prestretch. (**a**) *λ* = 0, (**b**) *λ* = 0.01, (**c**) *λ* = 0.02 and (**d**) *λ* = 0.03. The inset shows the stress distribution in the model under different prestrain conditions.

Applying prestretch strain to the model can realize active realtime control of BG under slight deformation. In order to study the effect of prestretch strain on BG, combined with the optimized geometric parameters, the plate thickness *t* = 1 mm, column radius *r*_1_ = 3.75 mm, diagonal hole radius *r*_2_ = 3.75 mm, periodic hole radius *r*_3_ = 3 mm and column height *h* = 7 mm are taken. Figure 7a is the band structure of the model when no prestrain is applied, (b)—(d) is the band structure of the model after applying an equal biaxial applied load, i.e., *λ*_*x*_ = *λ*_*y*_ = *λ*, to the matrix plate, with nominal strain *λ* values of 0.01, 0.02 and 0.03, respectively. We can learn from Fig. 7 that with the increase of nominal strain value, the BG changes show a certain regularity and a plurality of complete BGs are opened in the low-frequency range of 0–100 Hz. The insets in Fig. 7 show the stress distribution in the model under different prestrain conditions. Compared with the modal diagrams before and after applying prestretch strain in Fig. 7, it can be found that the geometric shape of the model is almost unchanged, but the surface stress distribution is not uniform.

**Figure 8:**
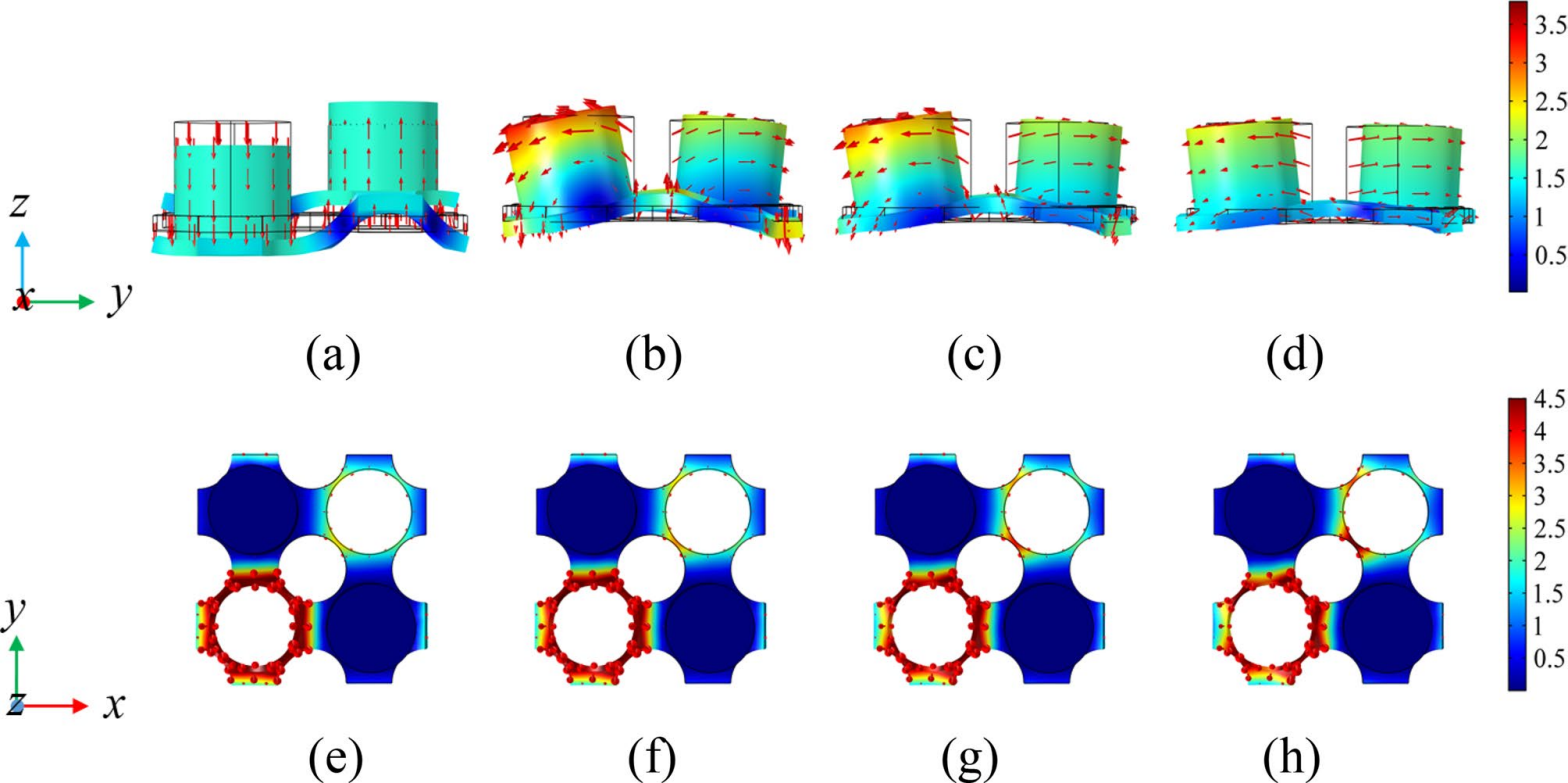
The eigenmodes shapes at the edges of the BG. Panels (**a**–**d**) correspond to the eigenmodes shapes at the point *Γ* of the sixth band when *λ* = 0, 0.01, 0.02 and 0.03, respectively. Panels (**e**–**h**) correspond to the eigenmodes shapes at the point *Γ* of the thirteenth band when *λ* = 0, 0.01, 0.02 and 0.03, respectively.

**Figure 9:**
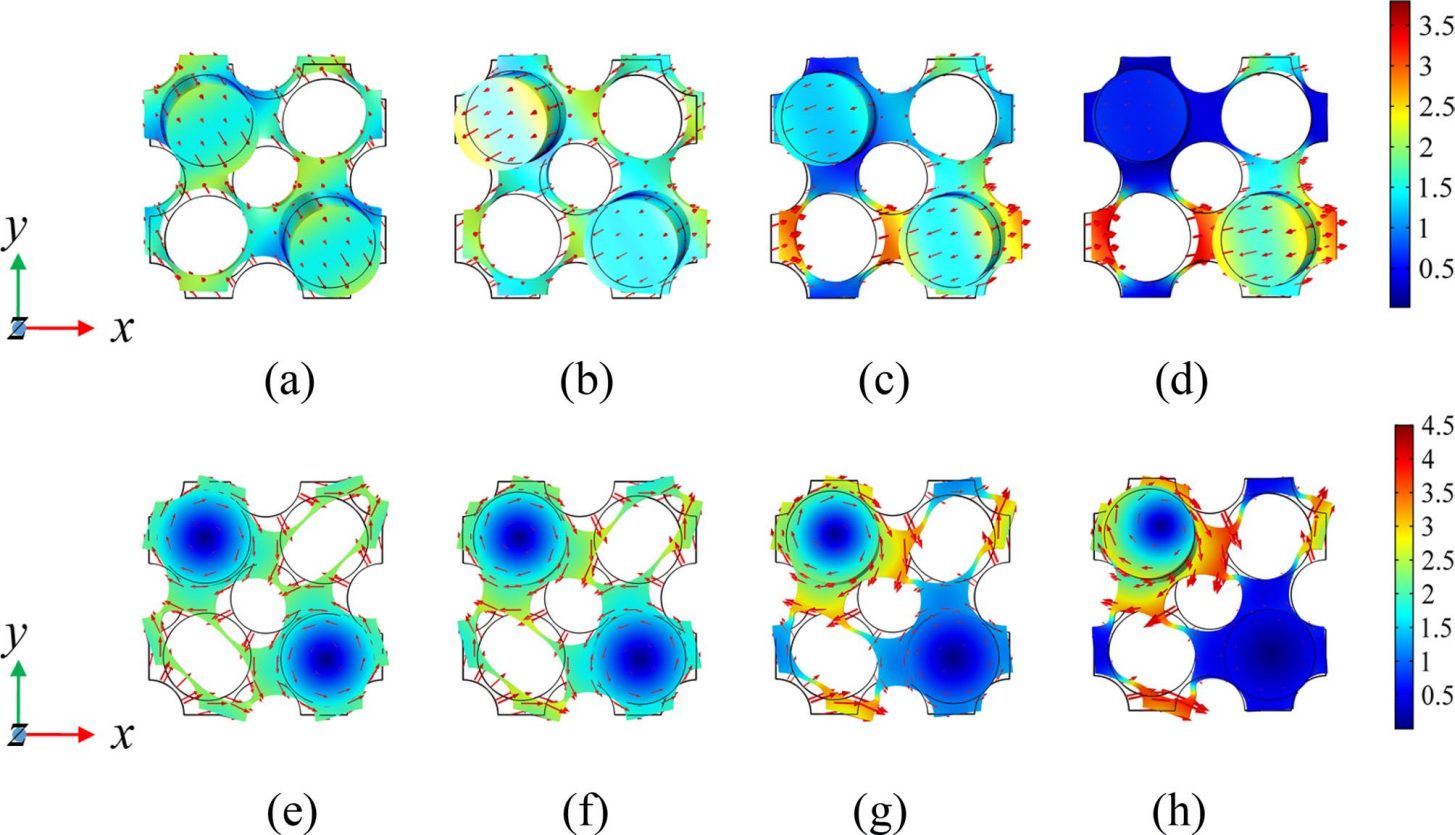
The eigenmodes shapes at the edges of the BG. Panels (**a**–**d**) correspond to the eigenmodes shapes at the point *Γ* of the seventh band when *λ* = 0, 0.01, 0.02 and 0.03, respectively. Panels (**e**–**h**) correspond to the eigenmodes shapes at the point *Γ* of the twelfth band when *λ* = 0, 0.01, 0.02 and 0.03, respectively.

To further analyze the change of BG in Figs. 7, 8 and 9 show the eigenmodes shapes of different bands in the band structure diagram under different prestretch strains at the high symmetry point *Γ*, where the points A-H correspond to the *Γ* points of the sixth and twelfth bands under different prestrain, respectively, points A_1_-H_1_ correspond to *Γ* points of the seventh and thirteenth bands under different prestrain, respectively. Observing Fig. 8a–d, it can be seen that the vibration mode of the structure changes from the out-of-plane translational resonance mode to the out-of-plane torsional resonance mode after the prestretch force is applied to the matrix plate and the vibration amplitude of the latter decreases obviously with the increase of the prestretch value, with the vibration is mainly concentrated in the column part. At this time, the plates at the junction of matrix plate and scatterers can be equivalent to springs, the rest of the vibration part of the matrix plate and the column are equivalent to mass blocks. According to the insets in Fig. 7, after prestretch strain is applied, the internal stress of the model is mainly concentrated in the part where the matrix plate is connected with the scatterer, and the internal stress value rises with the increase of external nominal strain, so the equivalent stiffness of the spring also increases. According to Eq. (5), the frequency of the sixth band rises with it, which is consistent with the change trend shown in Fig. 7. Figure 8e–h is the out-of-plane translational vibration mode of the plate, at which time the two cylinders are almost stationary. The vibration mainly concentrates in one corner of the matrix plate, so that the equivalent stiffness increases to a limited extent with increasing internal stress. In parallel, with the increase of the nominal strain value, the vibration gradually shows a trend of inverse localization and the equivalent mass of the vibration part increases, which leads to the decrease of the corresponding frequency of the thirteenth band. However, due to the increase of the equivalent stiffness to a certain extent, the decrease of the BG frequency is limited.

**Figure 10:**
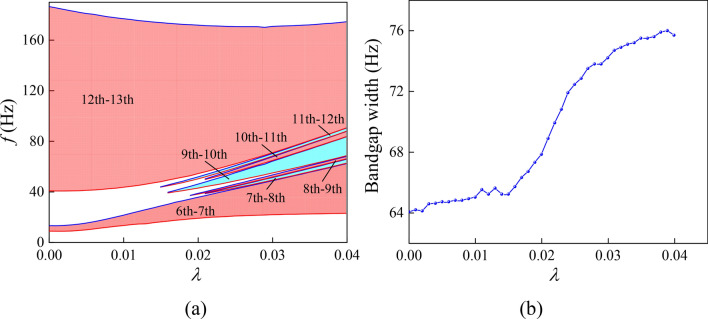
BG changes of PnC plate under different prestretch. (**a**) Evolution of BG with different nominal strain *λ*. (**b**) BG width in the range of 0–100 Hz with different nominal strain *λ*. The areas filled by red and blue in the schematic diagram represent the frequency range of BG under different nominal strain *λ*.

Based on the above analysis, it can be concluded that the optimization and active realtime control of low-frequency BG under small deformation can be realized by applying equal biaxial stretch strain to the model matrix plate. Due to the nonlinear behavior of silicone rubber, the effective stiffness of the equivalent spring varies greatly when a small prestrain is applied, resulting in a change in the resonant frequency of the corresponding mode. At the same time, when the effective mass of the vibration part of the model raises with the increase of the prestretch strain, the corresponding modal resonance frequency shows a downward trend. On the basis of above, the purpose of adjusting the low-frequency BG range can be achieved by changing the effective stiffness of the model material and the effective mass of the vibrating part. Observe the band distributed in the range of 0–100 Hz in Fig. 7, it can be seen that the increase of the prestretch strain value is beneficial to the flattening of the low-frequency band curve and finally forms a flat band. In order to study the causes of the flat band, the eigenmodes shapes corresponding to the *Γ* points of the seventh and twelfth bands under different prestrain are analyzed in Fig. 9. It can be seen from the Fig. 9 that with the increase of the prestrain value, the vibration of the modes corresponding to the two bands gradually concentrates on the lead column in the lower right corner as well as the upper left corner and the plates connected thereto, respectively, that is, the structural vibration mode gradually tends to the single eigenmode of the local structure. Therefore, the coupling frequency of the incident sound waves in all directions with the structure gradually tends to the resonance frequency of the local structure. The above analysis demonstrate that the localization effect of the model vibration becomes more and more obvious with the increase of the prestrain value *λ*. The vibration mode of the local harmonic oscillator is coupled with the traveling wave in the matrix and the traveling wave energy gradually decreases due to the coupling consumption, resulting in the decrease of the energy transfer speed of the corresponding frequency elastic wave. As such, the band curve corresponding to the relevant mode tends to be flat, finally opens a plurality of local resonance BGs between the seventh and the twelfth band, as shown in Fig. 10a. As can be seen from Fig. 10b, with the increase of strain value, the bandwidth in the range of 0–100 Hz basically shows an upward trend, which is consistent with the previous analysis results. The above results show that increasing the prestretch strain value in a certain frequency range is favorable to the widening of the low-frequency BG.

From the above, although the applied prestretch strain is small, the BG frequency is significantly adjusted in the low-frequency range and the BG variation trend shows certain regularity. In the process of pressure field control, the existence of column in plate-column structure is conducive to vibration modes in a certain low-frequency range tend to be localized, resulting in the bands tend to flatten and the opening of multiple BGs in the low-frequency range. Compared with the traditional regulation method of external load, the application of prestrain will not cause obvious structural deformation in the regulation process, so the application range is wider.

## Conclusions

By applying large mechanical load to the structure of soft PnCs, it provides convenience for BG control, but large deformation is not conducive to the practical application of PnCs and limits the application range of such PnCs. Alternatively, how to control the broadband noise between 20 and 200 Hz through external excitation also shows difficulties in the current research. We design a multivibrator soft PnC by introducing periodic holes into the traditional single-sided column PnC plate as well as analyzing the generation mechanism of the model’s low frequency BG and the influence of different geometric parameters on the BG. On this basis, the key geometric parameters for the design of model with low-frequency BG are presented and the realtime adjustable characteristic of the low-frequency BG under prestretch strain is further studied.

Compared with the traditional single-sided PnC plate, the starting frequency of the BG for novel PnC plate is lower and its bandwidth is wider. Due to the introduction of periodic holes, multiple vibrators appear in the structure and the generation of wide BG is the result of local resonance mode coupling of the multivibrators. Considering the in-plane and out-of-plane symmetry and stiffness of the structure, the BG can be optimized by changing the column height, the radii of diagonal holes and periodic holes. The optimized model obtained by changing the geometric parameters of the structure can generate a complete BG with a ratio of 92% in the low-frequency range of 0–450 Hz and the starting frequency of the widest BG is as low as 32.3 Hz.

Applying equal biaxial prestretch strain in *x* and *y* directions to the designed PnC plate, it is feasible to implement active realtime control of low frequency BG under small deformation. In parallel, prestretch strain can open new low-frequency BGs and the BGs’ frequency range widens with the increasing strain. After analyzing the corresponding modes, it is found that the localization degree of the unit cell vibration increases with the rises of the prestrain value, thus the band curve tends to be flat, eventually leading to the opening of the new local resonance BGs. The results provide a reference for the optimization of the BG of plate-column type PnCs and also offer ideas for improving the realtime adjustability of the PnCs’ low-frequency BG in complex acoustic vibration environments.

## Data Availability

The data that support the findings of this study are available from the corresponding author upon reasonable request.
